# Heart Failure in North America

**DOI:** 10.2174/1573403X11309020006

**Published:** 2013-05

**Authors:** John E. A Blair, Mark Huffman, Sanjiv J Shah

**Affiliations:** San Antonio Military Medical Center, San Antonio, TX; Northwestern University Feinberg School of Medicine, Chicago, IL, USA

**Keywords:** Heart failure, epidemiology, regions, North America.

## Abstract

Heart failure is a major health problem that affects patients and healthcare systems worldwide. Within the continent of North America, differences in economic development, genetic susceptibility, cultural practices, and trends in risk factors and treatment all contribute to both inter-continental and within-continent differences in heart failure. The United States and Canada represent industrialized countries with similar culture, geography, and advanced economies and infrastructure. During the epidemiologic transition from rural to industrial in countries such as the United States and Canada, nutritional deficiencies and infectious diseases made way for degenerative diseases such as cardiovascular diseases, cancer, overweight/obesity, and diabetes. This in turn has resulted in an increase in heart failure incidence in these countries, especially as overall life expectancy increases. Mexico, on the other hand, has a less developed economy and infrastructure, and has a wide distribution in the level of urbanization as it becomes more industrialized. Mexico is under a period of epidemiologic transition and the etiology and incidence of heart failure is rapidly changing. Ethnic differences within the populations of the United States and Canada highlight the changing demographics of each country as well as potential disparities in heart failure care. Heart failure with preserved ejection fraction makes up approximately half of all hospital admissions throughout North America; however, important differences in demographics and etiology exist between countries. Similarly, acute heart failure etiology, severity, and management differ between countries in North America. The overall economic burden of heart failure continues to be large and growing worldwide, with each country managing this burden differently. Understanding the inter-and within-continental differences may help improve understanding of the heart failure epidemic, and may aid healthcare systems in delivering better heart failure prevention and treatment.

## INTRODUCTION

Heart failure (HF) is a major global public health problem. Worldwide recognition and treatment of acute myocardial infarction (MI) and infection-related heart disease, such as rheumatic heart disease, has improved over time while the epidemics of obesity, diabetes mellitus (DM), and metabolic syndrome continue to soar in magnitude, thereby setting the stage for the resultant epidemic of chronic cardiovascular disease (CVD) and HF. HF is the final common pathway for most forms of CVD, and is therefore a heterogeneous syndrome and not a disease per se. While the heterogeneity of underlying etiologies of HF has been the focus of much prior investigation, continental and country-specific differences in HF are additional, critically important (though often underappreciated) reasons for HF heterogeneity. Differences in economic development, genetic susceptibility, cultural practices, and trends in risk factors all contribute inter-continental and within-continent differences in HF. The purpose of this review is to provide an overview of HF in North America, by highlighting the epidemiology, risk factors, management strategies, costs, and future challenges of HF in the United States (U.S.), Canada, and Mexico.

## HEART FAILURE EPIDEMIOLOGY

The U.S. and Canada, industrialized countries with similar culture and geography, have advanced economies and infrastructure. During the transition from rural to industrial countries over the past century, major causes of death and disability have shifted from predominantly nutritional deficiencies and infectious diseases, to degenerative diseases such as CVD, cancer, and diabetes. This shift has been referred to as “the epidemiologic transition.”[1] Some risk factors for HF such as MI and untreated blood pressure have decreased, while increases in other risk factors such as obesity and DM, coupled with the aging population, have resulted in increased incidence of HF. Advances in treatment of HF, particularly neurohormonal blockade and device therapy in the setting of reduced left ventricular (LV) ejection fraction (EF), have improved HF-associated survival, leading to increased prevalence of chronic HF [2]. 

Mexico, on the other hand, has a less developed economy and infrastructure. It is undergoing urbanization in many of its cities, but this process is not homogenous throughout the country. Life expectancy has improved during this urban transformation, resulting in less death from infectious disease or nutritional deficiencies, but high-fat and high-sugar diets, cigarette smoking, and sedentary lifestyles have replaced behaviors traditional to the region [3, 4]. The emergence of CVD and other chronic diseases are relatively new to Mexico, and its downstream effect on HF has yet to be determined. 

There were approximately 5.7 million U.S. Americans ≥ 20 years of age (2.7% prevalence) living with HF, and 670,000 new cases ≥ 45 years of age per year in 2008 in a population of approximately 304 million [2]. The incidence of new HF hospitalizations in the U.S. tripled between 1979 and 2004 [5], with a slightly higher prevalence in men than women and a heavy predominance with advancing age [2]. Similar to the United States, there were approximately 500,000 Canadians living with HF (1.5% prevalence), with an annual incidence of 50,000 new cases in a population of approximately 32.7 million in 2006, also with a predilection for advanced age [6]. As both U.S. and Canadian populations age, and survival from CVD improves, the overall burden of HF is expected to increase over time. The prevalence and incidence of HF in Mexico is not known.

## HEART FAILURE RISK FACTORS

Trends in risk factors help explain the prevalence and incidence of HF within a region. The following section will discuss the major risk factors for HF and how they vary between the countries in North America.

### Coronary Heart Disease

Coronary heart disease (CHD) carries the highest relative risk among conventional risk factors for HF [7-9]. In an analysis of 13,643 participants in the National Health and Nutrition Examination Survey (NHANES)-I Epidemiologic Follow-up Study, the presence of CHD was the most strongly associated risk factor for HF (relative risk [RR] 8.11, 95% confidence interval [CI] 6.95-9.46, Table **1**) [9]. It is estimated that 8% of men and 18% of women 45-64 years of age and 20% of men and 23% of women ≥ 65 years of age who have had an MI will develop HF within 5 years [10]. Ischemia was identified as a precipitating factor for HF hospitalization in 14.7% of patients enrolled in the Organized Program to Initiate Lifesaving Treatment in Hospitalized Patients with Heart Failure (OPTIMIZE-HF) registry, second only to pneumonia or respiratory process, and was independently associated with a worse in-hospital (odds ratio [OR] 1.20, 95% CI 1.03-1.40) and post-discharge (OR 1.52, 95% CI 1.20-1.93) mortality [11].

In the U.S., analysis of the National Hospital Discharge Survey (NHDS) from 1979-2005 demonstrated a decline in the hospitalization rate for MI in the U.S. since 1996 [12]. In addition, in-hospital case-fatality has declined during this period as the use of reperfusion therapies increased. Improved survival from MI may increase the pool of patients with myocardial damage, resulting in increased incidence of HF. Indeed, an analysis of 676 Framingham Heart Study participants age 45-85 years of age who developed their first MI between 1970 and 1999 demonstrated an increase in 30-day incidence of HF after MI from 10.0% in 1970-1979 to 23.1% in 1990-1999 (P for trend = 0.003), during which time MI mortality declined from 12.2 to 4.1% (P for trend = 0.02) [13]. Similar results were demonstrated for 5-year outcomes (increase in HF incidence 27.6% to 31.9% [P for trend = 0.02], and decrease in MI mortality from 41.1% to 17.3% [P for trend <0.0001]). The trend for increased incident HF in the face of improved post-MI survival was also demonstrated in the 10,440 patients during the time period of 1975-2001 in the Worchester Heart Attack Study [14]. 

**Fig. (1):**
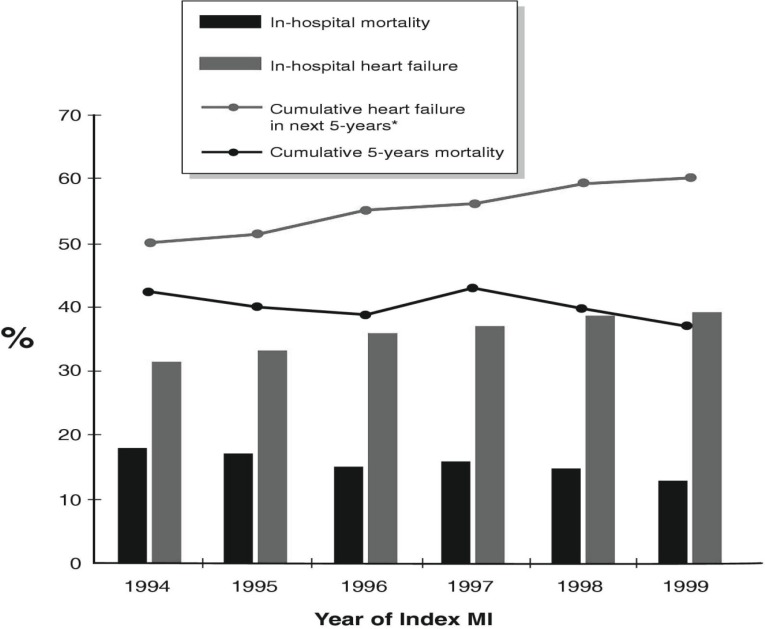
Temporal trends in mortality rate and development of heart failure in 7,733 patients ≥ 65 years of age after first myocardial infarction in Alberta, Canada. Black bars – in-hospital mortality rate; Grey bars – in-hospital heart failure rate; Grey line – cumulative heart failure in the next 5 years; black line – cumulative 5-year mortality. Reprinted with permission [15].

In Canada, the Alberta Elderly MI cohort combined 5 separate databases maintained by the Alberta Health and Wellness to examine 7,733 patients ≥ 65 years of age with their first MI and at least 5 years of follow-up (Fig. **1**) [15]. This Canadian study demonstrated that between 1994 and 1999, 5-year mortality rate after MI decreased by 28% and the 5-year rate of HF development increased by 25%, similar to the pattern observed in the United States.

Data on survival from MI in Mexico does not exist in the literature; however, reperfusion and survival trends appear to be similar to those occurring in the U.S. and Canada [16]. Prevalence of ischemic CHD appears to be on the rise in countries in Latin America and other developing countries [17]. The combination of improved survival from MI and the increasing prevalence of CHD in Latin American countries like Mexico suggest that the global burden of HF from CHD will most likely continue to increase [16]. CHD continues to be the most important risk factor for HF in industrialized countries, mostly as a result of modern reperfusion and pharmacological therapies resulting in survival from MI, and is becoming a more important risk factor in developing countries where overall CHD prevalence is rising as a result of longer life expectancy and emergence from poverty. 

### Hypertension

At the individual patient level, the risk of developing HF is lower in the setting of hypertension (HTN) compared with MI. However, because of its high population prevalence, HTN carries a high population-attributable risk for HF, accounting for 39% of cases in men and 59% in women, according to analysis of 5,143 patients 40-89 years in age in the Framingham Heart Study and Offspring Study [18]. The Cardiovascular Health Study (CHS), composed of 5,888 elderly patients, found a lower population-attributable risk of HF for HTN of 12.8%, second to CHD (13.1%) [7], and the (NHANES)-I Epidemiologic Follow-up Study found a population-attributable risk of HF for HTN was 10.6%, third to CHD (61.6%), and tobacco smoking (17.1%) [9]. The differences in the relative population-attributable risks may be due to differing patient populations studied and differing study methods. Lifetime risk of HF is increased with more severe elevations in blood pressure [8], and the combination of HTN and HF carries a poor prognosis if left untreated [18].

In the U.S., it is estimated that one in three adults have HTN, [10) and the prevalence is projected to grow by an additional 9.9% from 2010 to 2030 [19]. Analysis of 42,856 NHANES adults > 18 years of age surveyed between 1988-1994 and 1999-2008 demonstrated an increase in the rate of HTN from 23.9% (95% CI 22.7-25.2%) in the 1988-1994 period to 28.5% (95% CI 25.9-31.3%) in the 1999-2000 period, but this rise stabilized between the 1999-2000 and 2007-2008 periods (29.0, 95% CI 27.6-30.5%) [20]. The same study demonstrated an increase in the awareness and treatment of HTN, along with an increase in percent controlled from 27.5% (95% CI 25.6-29.1%) in the 1988-1994 period to 50.1% (95% CI 46.8-53.5%) in the 2007-2008 period. 

In Canada, analysis of 28,352 adults in three surveys demonstrated a lower and stable prevalence of HTN during a similar time period as the U.S. NHANES between 1992-2009 at 19.7-21.6% [21]. Control of HTN in Canada has improved much more dramatically than in the U.S., from 13.2% (95% CI 10.7-15.7%) in 1992 to 64.6% (95% CI 60.0-69.2%) in 2009, accompanied by similar improvements in awareness and treatment. [21]. The marked improvement in HTN control in Canada compared to the U.S. may be due to the higher percent increase in antihypertensive prescription in Canada compared to the U.S. starting in the late 1990s [22, 23], the effectiveness of the Canadian Hypertension Education Program [24], and the differences in reimbursement schedules for drugs in the different countries. 

A national survey of 14,657 people 20-69 years of age in Mexico between 1992-1993 demonstrated a crude HTN rate of 28.1% in women and 37.5% in men, similar to rates in the contemporary U.S. However, HTN awareness and control rates were both very low (28% and 22%, respectively) [25]. Nationally-representative data on trends in HTN prevalence, treatment, and control are lacking in Mexico. In North America, Canada has the lowest rates of HTN and the largest change in HTN control over the last several decades, likely due to national initiatives designed to improve awareness and treatment of this disease, while rates in the U.S. and Mexico are higher. Recent modeling of the effect of a modest reduction in dietary salt on reduction has suggested a significant and cost-effective reduction in HTN and CHD outcomes [26]. It remains to be determined whether national initiatives to reduce dietary salt intake would result in reduction in HTN, CHD, and incident HF. 

### Diabetes

Insulin resistance and DM are major risk factors for the development of HF [27, 28]. The presence of insulin resistance impacts left ventricular (LV) remodeling [29, 30] and has been implicated in overt systolic [31] and diastolic dysfunction [32]. DM was a major risk factor for the development of HF in the analysis of 13,643 men and women in the NHANES I Epidemiologic Follow-up Study (RR 1.83, 95% CI 1.27-2.63 for men and RR 1.83, 95% CI 1.38-2.41 for women, Table **1**) [9]. The combination of DM and HF portends a grim prognosis. Analysis of 665 subjects in Olmsted county between 1979 and 1999 with HF demonstrated that those with both DM and HF were younger, had a greater body mass index, and had a lower LVEF than those without DM [33]. These subjects had a greater risk of death than those without DM, independent of age, sex, creatinine clearance, ejection fraction, and year studied (RR 1.33, 95% CI 1.07-1.66) [33]. 

**Fig. (2):**
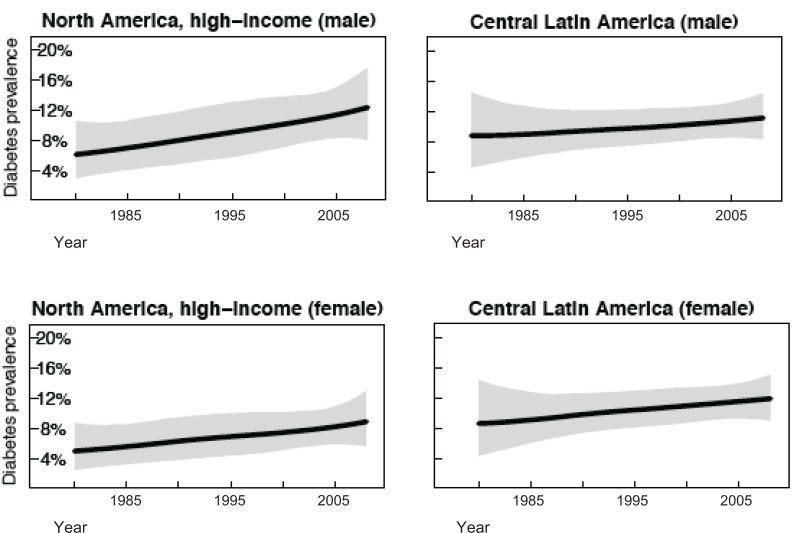
Trends in age-standardized diabetes by region, 1980-2008. Reprinted with permission [34].

In the U.S., 11.3% of people ≥ 20 years of age have DM [2]. Analysis of health examination surveys and epidemiological studies including over 370 country-years and 2.7 million participants demonstrated that from 1980-2008, the prevalence of DM has doubled in U.S. men (6.1% [95% CI 2.9- 2.9-10.7%] to 12.6 [95% CI 8.1-18.1]) and nearly doubled in women (5.1% [95% CI 2.4-8.9%] to 9.1% [95% CI 5.7-13.3%]), both in a linear manner over time (Fig. **2**) [34]. Similar trends are seen in Canadian men (7.6 [95%CI 1.1-21.2%] to 10.9% [95% CI 2.5-26.3%]) and women (5.5% [95% CI 0.7-16.7%] to 8.3% [95% CI 1.6-20.9%]). The prevalence of DM in Mexico in 1980 was much higher than in the U.S. and Canada, with a more gradual linear trend over time to 2008 (8.9% [95% CI 3.1-17.3%] to 13.2 [95% CI 8.6-18.8%] for men and 9.4% [95% CI 3.4-17.7%] to 14.9% [95% CI 10.2-20.4%] for women). Trends in DM in North America indicate that although Mexico has the highest prevalence and a steep upward trend currently, the rate of DM in the U.S. is increasing at a faster rate and will soon surpass that of Mexico.

### Other Risk Factors

Overweight and obesity are known risk factors for the development of HF [9, 35]. Analysis of 5,881 subjects in the Framingham Heart Study demonstrated a two-fold increase in the risk for HF in obese (body mass index [BMI] ≥30 kg/m^2^) compared to those with normal-weight (BMI 18.5-24.9 kg/m^2^) participants (HR 2.04, 95% CI 1.59-2.63), as well as an increased risk for HF by 4% in men (HR 1.04, 95% CI 1.00-1.07) and 7% in women (HR 1.07, 95% CI 1.04-1.10) for every 1 kg/m^2^ increase in BMI, after adjustment for established risk factors [36]. In the U.S., 35.5% of adult men and 35.8% of adult women were obese in 2009-2010, with no significant change from 1999-2010 in women, but gradual increase in obesity prevalence for men (adjusted OR 1.04 per year, 95% CI 1.02-1.06), with an apparent leveling off in the most recent 2 years compared to the previous 6 years, according to NHANES data [37]. Data from the Canadian Health Measures Survey 2007-2009 revealed a prevalence of obesity in Canada of 24.3% in adult men and 23.0% in adult women, significantly lower than the U.S. NHANES 2007-2008 survey of 32.6% in adult men and 36.2% in adult women, and similar to the U.S. NHANES 1988-1994 survey [38]. However, over the past 20 years, increases in obesity prevalence were similar in both countries for both sexes (increase by 12.0% and 10.3% in U.S. adult men and women, respectively, versus 10.3% and 8.2% in Canadian adult men and women, respectively [38]. The Mexican National Health Survey in 2000 determined the prevalence of obesity to be 19.4% in men and 29.0% in women, similar to those in the U.S. NHANES 1988-1992 survey, and higher than a prior Mexican survey in 1988-1992, indicating that Mexico is not far behind the U.S., and probably ahead of Canada in obesity prevalence [39]. 

Active cigarette smokers had a 49% higher risk of developing HF than non-smokers (RR 1.49, 95% CI 1.30-1.70) in the NHANES-I Epidemiologic Follow-up study [9]. In subjects with LVEF <35% enrolled in the Study Of Left Ventricular Dysfunction (SOLVD) Prevention and Intervention trials, current smoking was associated with a 39% increased risk of death or hospitalization for HF or MI (RR 1.39, 95% CI 1.23-1.57), compared to subjects who have never smoked [40]. There was no significant difference in outcomes between ex-smokers and never-smokers, suggesting a potential benefit to smoking cessation in patients with established LV systolic dysfunction. Tobacco use is slowly declining in many affluent countries, whereas it is increasing in developing countries [41]. In the U.S., there was a decline in prevalence of adult cigarette smokers from 24.1% to 19.3% from 1998-2010, with an apparent plateau between 2005-2009, and small but significant decline between 2009-2010 [10]. Prevalence in Canada is slightly lower than in the U.S., with an overall decline in smoking rates in Canadians ≥ 15 years of age from 25% in 1999 to 18% in 2009 [42]. An international household survey including 13,617 Mexicans between 2008-2010 revealed a tobacco smoking rate of 24.8% in men and only 7.8% in women ≥ 15 years of age, a rate higher than that of the U.S. for men, but much less for women [43]. 

The presence of valvular heart disease increases the risk of HF by 46%, with higher risk in men (RR 1.74, 95% CI 1.31-2.31) compared to women (RR 1.36, [95% CI 1.00-0025; CI 1.00-1.84]) in the NHANES-I Epidemiologic Follow-up Study (Table **1**) [9]. The etiology of valvular heart disease has shifted from rheumatic to degenerative in industrialized countries, whereas rheumatic heart disease remains an important cause in developing countries [44]. Pooled echocardiographic and clinical data from 11,911 subjects in 3 NHLBI studies determined an age-adjusted prevalence of moderate to severe valvular heart disease of 2.5% [95% CI 2.2-2.7%] in the U.S., with a sharp increase in prevalence in subjects ≥ 65 years of age, suggesting that valvular heart disease and its consequent HF present primarily in the elderly in industrialized countries such as the U.S. and Canada [45]. Review of global prevalence of rheumatic heart disease estimates 1.3 cases per 1000 in Latin America, and only 0.3 per 1000 in established market economies [46]. 

## RACIAL AND ETHNIC DIFFERENCES IN HEART FAILURE

The U.S. and Canada are comprised of high proportions of ethnic minorities relative to other countries. According to the 2010 U.S. and 2006 Canadian Censuses, 36.3% of the U.S. population and 16.2% of the Canadian population is comprised of ethnic minorities. [47, 48]. The demographic makeup of these countries, however is very different. In the U.S., ethnic minorities are mostly Hispanic, black, other, or Asian (16.3%, 12.6%, 6.2%, and 4.8% of the population, respectively), with a very small percentage Native American and Alaskan Native (0.9%). Canadian ethnic minorities are dominated by Asian ethnic groups (South Asian, Chinese, and other Asian representing 4.0%, 3.9%, and 3.2% of the population, respectively), followed by a large population of Aboriginals (3.8% of the population), and relatively small black and Hispanic populations (2.5% and 1.0% of the population, respectively). The prevalence of HF risk factors among different ethnic group influences the incidence and type of HF in distinct groups. In addition, disparity in access to care influences quality of care for HF, severity of presentation for AHFS, and overall outcomes in underserved ethnic groups. The following section will evaluate racial and ethnic differences in HF in the U.S. and Canada. No such data exist for Mexico.

### The Hispanic Population in the United States:

Hispanic Americans lead all ethnic groups in the U.S. in numbers and rate of population growth, currently comprising 16.3% of the population [47]. The majority of Hispanics in the U.S. are Mexican (63%), followed by Puerto Rican (9.2%), Cuban (3.5%), and Dominican (2.8%). According to recent data from the Multi-Ethnic Study of Atherosclerosis (MESA), a cohort study of 6,814 participants of 4 ethnicities (white, African American, Hispanic, and Chinese American) in 6 communities, Hispanic Americans had the second-highest incidence of HF at 3.5 per 1,000 person-years, second to African Americans at 4.0 per 1,000 person-years at a mean follow-up of 4.6 years [49]. White and Chinese Americans had lower incidence rates of 2.4 and 1.0 per 1,000, respectively. MESA and other data have shown that compared to other ethnic groups with HF, Hispanics rank highest in rate of DM (tied with African Americans), dyslipidemia, and renal dysfunction, second-highest in rate of obesity (second to African Americans), percentage uninsured (second to Chinese Americans), and second-youngest in age of presentation for HF (second to African Americans), placing this population at high risk for the development of HF [49, 50]. In addition to high rates of DM and HTN, observational studies have shown that glycemic [51] and blood pressure [52] control are worse in Hispanics compared to non-Hispanic whites. MESA data have demonstrated that Hispanics have intermediate rates of MI and coronary calcium scores [49]. Increased LV mass and wall thickness measured by magnetic resonance imaging were completely attributable to subclinical atherosclerosis and HTN in multivariate analysis for non-Hispanic whites, but not for Hispanics, suggesting a greater contribution of DM to LV abnormalities in Hispanics [53]. In addition to the above risk factors for HF, the incidence of rheumatic heart disease remains high for Latin American immigrants, whose prevalence of this disease mimics that of their native country. Of all ethnic groups, Hispanic have the lowest medical insurance access [54] and are the most likely to have no usual place of care [55]. 

Hispanics have higher rates of rehospitalization for HF than non-Hispanic whites, as seen in two large cohort studies using the Medicare Provider Analysis Record and a California discharge database [50, 56]. Paradoxically, Hispanic patients with HF have lower in-hospital and short-term mortality rates [50, 56]. Both the rehospitalization rates and mortality rates for Hispanics are intermediate between African Americans and non-Hispanic whites. Hispanic HF patients also seem to enjoy more significant improvement in health-related quality of life over time than other ethnic groups [57]. The available data on HF in the U.S. Hispanic population depict this group as a particularly high-risk population for HF that has significant barriers to care, which both contribute to the high HF hospitalization and rehospitalization rate. Despite this, this population currently has favorable mortality and quality of life compared to other ethnic groups. As the Hispanic population continues to grow, there will be many challenges in reducing HF disease burden, morbidity, and mortality in this vulnerable population. 

### The African American Population in the United States

African Americans make up 12.6% of the population in the United States. In the U.S., black individuals have a higher prevalence of HF than members of other ethnicities, and present at younger ages [58]. The Coronary Artery Risk Development in Young Adults (CARDIA) study prospectively assessed the incidence of HF over 20 years in 5,115 African Americans and whites of both sexes, ages 18-30 years [59]. This study demonstrated that early-onset HF affected African American men and women 20 times more than that of white men and women, partially attributed to the development of antecedent of HTN, obesity, chronic kidney disease, and development of depressed LVEF. Follow-up of the Atherosclerosis Risk in Communities (ARIC) study, a population-based study of subjects aged 45-64 at entry in 4 United States Communities initiated between 1987 and 2002, demonstrated that incident HF was lowest in white women at 3.4 per 1,000 person-years, followed by 6.0 for white men, 8.1 for black women, and 9.1 for black men, an effect that was partially accounted for by higher prevalence of risk factors such as CHD, DM, and HTN in black men and women [60]. In MESA, African Americans had the highest incidence of HF at 4.6 per 1,000 person-years, and had the highest rates of obesity, tobacco smoking, DM (tied with Hispanics), and HTN, and LV mass index among all ethnic groups studied [49]. Adding the traditional risk factors of DM and HTN to models including ethnicity eliminated differences in incident HF between African Americans and white Americans, whereas age, sex, obesity, tobacco smoking, and education level did not result in significant changes in the magnitude of this association. Adding household income, daily caloric and trans-fat intake, use of ACE-inhibitors and calcium channel blockers had very similar effects to the effects of adding DM or HTN. Interim MI was not present in 75% of incident HF cases among African Americans, 60% among whites, and 58% among Hispanics (P=0.06), and adding coronary artery calcification and interim MI to models using DM and HTN increased the association between African American race and incident HF. The MESA data suggest potential mechanisms for the racial disparity in incident HF. Although differences in traditional risk factors may account for much of this disparity, socioeconomic factors may also play a role, as may differences in ventricular remodeling in the presence of ischemic heart disease or after MI. 

Although there has been suggestion that there are disparities in quality of care by race in the community [61], large retrospective database analyses using objective criteria for HF quality such as ACE-inhibitor use and LVEF in Medicare and Veterans Affairs beneficiaries demonstrated comparable quality of care between African American and white patients [62, 63]. There are significant differences in the characteristics and outcomes of African Americans presenting with acute HF. Retrospective chart review of 753 consecutive patients admitted with HF at a single Veterans Administration facility between 1997-1998 was conducted, with treatment-seeking delay measured as the time from worsening of HF symptoms to the time of presentation to the hospital [64]. The mean treatment-seeking delay was significantly longer for African Americans (3.2 days) versus Caucasians, Asians, and Hispanics (2.8, 2.9, and 2.8 days, respectively, P=0.019), a trend that remained significant after multivariate analysis. Analysis of 29,862 African American patients and 105,872 white patients hospitalized for HF between 2001 and 2004 in the Acute Decompensated Heart Failure National Registry (ADHERE) database demonstrated that compared to white patients, African American patients were younger (mean age 63.5 versus 72.5 years), less likely to have an ischemic origin of HF (30.0% versus 56.1%), more likely to have a LVEF <40% (58.4% versus 49.7%), more likely to have DM, HTN, and obesity, more likely to have renal dysfunction on presentation; however, African Americans had higher initial blood pressure and a narrower QRS complex on electrocardiogram [65]. Despite presence of higher comorbidities, lower LVEF, and worse renal function, African Americans had lower rates of in-hospital death (2.8% versus 4.5%) compared to white patients, which persisted after adjustment for major covariates in the non-ischemic subgroup but not the ischemic subgroup. 

Long-term mortality and rehospitalization rates after hospitalization for HF were analyzed in a nationwide U.S. sample of 29,732 Medicare beneficiaries hospitalized with HF between 1998-1999 [62]. African American patients had higher rates of readmission within 1 year of discharge (68.2% versus 63.0%, P<0.001) but had lower 30-day (6.3% versus 10.7%, P<0.001) and 1-year (31.5% versus 40.1%, P<0.001) mortality rates compared to white patients. These relative differences were maintained after multivariate analysis. In the ARIC study, which followed patients for up to 15 years after initial HF hospitalization, there was no difference in age-adjusted 30-day and 1-year case fatality rate between African American and white subjects, however at 5-years, African Americans demonstrated a significantly greater case fatality compared with their white counterparts for both men (P=0.02) and women (P=0.03), indicating a divergence of curves over time [66]. In SOLVD, in which patients with asymptomatic (in the prevention trial) or symptomatic (in the treatment trial) LV systolic dysfunction were randomized to enalapril or placebo, and overall mortality rates were 8.1 and 5.1 per 100 person-years for African Americans and whites, respectively after a mean follow-up of 34 months in the prevention trial, and 16.7 and 13.4 per 100 person-years, respectively after a mean follow up of 32 months in the treatment trial [67]. In SOLVD, the higher rate of all-cause death, death from pump failure, and the combined endpoint of death from any cause or hospitalization for HF in African Americans compared to whites was maintained after adjustment for major covariates, suggesting that LV dysfunction in African Americans progresses more quickly than in whites. Taken together, African Americans with HF or LV systolic dysfunction appear more susceptible to deterioration of LV function over time, which translates to worse rehospitalization rates and long-term mortality despite apparently better short-term mortality compared to their white counterparts. 

### The South Asian Population in Canada: 

The largest and fastest-growing ethnic minority group in Canada is the South Asian population (4.0% of the population), representing people from India, Pakistan, Bangladesh, Sri Lanka, Nepal, Afghanistan, Bhutan, and Maldives. This highly populated region has the second-largest proportion of the burden of cardiovascular diseases globally compared to other regions [3]. Ischemic heart disease is particularly prevalent and presents at younger ages in subjects living in South Asian countries compared to other countries, likely due to the prevalence of important risk factors such as abdominal obesity, diabetes, and tobacco smoking [68]. The higher incidence and younger age at presentation for MI have been observed in recent epidemiological surveys in Canada [69, 70]. Although short-term mortality after MI was similar in South Asian and white Canadians, the long-term mortality was lower for South Asian Canadians, which may influence the prevalence of HF from ischemic heart disease (IHD) in this population [70]. A retrospective case-control series of 553 South Asian patients and 553 non-South Asian patients presenting with acute MI to two Toronto-area community hospitals from 1994 to 1999 demonstrated that South Asians presented almost one hour later, and had more extensive coronary disease as evidenced by the need for urgent coronary artery bypass grafting but had similar in-hospital survival compared to non-South Asians. Despite more severe coronary artery disease and later presentation, South Asians have a similar survival after MI, which may increase the risk of HF from IHD. 

A review of 887 consecutive patients admitted with a primary diagnosis of HF from the two Toronto-area community hospitals between 1997 and 1999, of which 88 (12%) were identified as South Asian and 728 (88%) were identified as non-South Asian Caucasian was performed [72]. Compared to Caucasians, South Asians presented at a younger age (69.1 versus 75.1 years, P<0.001), had a lower body mass index (24.4 versus 26.7 kg/m^2^, P=0.003; despite similar height), were more often diabetic (57% versus 39%, P<0.001), were less likely to be current or former smokers (24% versus 41%, P=0.001), and had similar rates of hypertension, hyperlipidemia, MI, and prior HF presentation. Etiology was similar between South Asians and Caucasians (ischemic heart disease 48-49%, valvular heart disease 5-7%), as was ejection fraction (40-42%). Upon presentation, serum sodium level was lower in South Asians (135 versus 137 mmol/L, P=0.002) than in Caucasians, but other markers of HF severity such as serum creatinine, blood pressure, and heart rate were similar between groups. Use of intravenous diuretics, inotropes, vasodilators, ACE-inhibitors/ARBs, beta-blockers, and statins were similar between groups, and in-hospital procedures were infrequent and similar between groups. Overall unadjusted and adjusted in-hospital mortality was similar between groups. Considering the data from MI and HF in the Canadian South Asian population, it appears that this is a particularly high-risk group of patients susceptible to HF from CHD. Future efforts should be aimed at elucidation of mechanisms by which IHD develops in this population and prevention of IHD and early MI.

### The Chinese Population in Canada:

The Chinese population is the second largest ethnic minority group in Canada comprising 3.9% of the population. Mandarin is the third most commonly spoken language in Canada [73]. This population continues to maintain close family links and emphasize family values, which both play an important role in medical decision making. A recent large multilingual telephone survey of 1004 ethnic Chinese subjects in Toronto and Vancouver in 2004 demonstrated that 32% and 40% of responders could not name at least one symptom of heart attack or stroke, respectively, and 32% and 35% named at least one incorrect symptom of heart attack and stroke, respectively [74]. This lack of knowledge and above-mentioned ethnocultural factors may confound management of Chinese Canadians with HF. 

According to a recent review of the literature, the etiology of HF in the Chinese population has shifted significantly since 1980 to 2000 away from valvular/rheumatic heart disease, towards ischemic heart disease and HTN, although valvular/rhematic heart disease was still the etiology of 9-21% of cases in 2000 [75]. Recent analysis of 200 consecutive Chinese patients admitted to a Hong Kong hospital with signs and symptoms of HF demonstrated with echocardiography that 12.5% had significant heart disease, and 66% had a normal left ventricular ejection fraction (LVEF >45%), likely due to the high number of elderly and hypertensive patients [76]. In Canada, death from IHD in the Chinese population is approximately half as prevalent in men and women compared to Caucasian or South Asian populations according to analysis of the Canadian Mortality Database from 1979-1993. [77) A chart review of from a tertiary care outpatient cardiology clinic in Toronto between 1994 and 1999 demonstrated older age, lower rates CHD, multivessel CHD, and HF in Chinese, and higher rates of valvular heart disease compared to Caucasian patients [78]. In the patients with HF, there were more Chinese patients with normal LVEF (>40%) compared to Caucasian patients. The Chinese population in Canada remains a challenge for management due to their relative limited insight on their disease status and their differing risk factors and type of HF. 

### The Black Population in Canada

The black population comprises 2.5% of the Canadian population. The majority of black individuals in Quebec are of Hatian and Central African descent and speak primarily French, while black individuals in other parts of Canada speak English [73]. Much of the data regarding differences in HF between black and non-black individuals exists in the literature from the United States and were discussed earlier. According to analysis of five cross-sectional surveys between 1996 and 2007 in Ontario, black subjects had the highest prevalence of two or more measured cardiovascular risk factors (smoking, obesity, DM, and HTN) among four ethnic groups studied, but paradoxically had a low prevalence of heart disease (3.4%) compared to the other groups (Caucasian 5.0%, South Asian 5.2%, Chinese 3.2%) [79]. 

### The Aboriginal Population in Canada:

The Aboriginal population represents 3.8% of Canada’s population and is comprised of First Nations, Métis, and Inuit people with more than one-half living off-reservation. Aboriginal people are at a disadvantage to non-Aboriginal people in that they are almost four times more likely to live in a crowded dwelling, three times as likely to live in a dwelling in need of major repairs, and have a 7.4 and 5.2 year shorter life expectancy for men and women, respectively [73]. Analysis of the Canadian Institute for Health Information database of all hospital admissions in Ontario between 1981-1997 was performed to assess trends in admission rates for IHD over the 17-year period between Aboriginal and non-Aboriginal populations [80]. This analysis demonstrated a progressively rising rate of IHD admissions from a nadir of 76 per 10,000 persons (95% CI 57-95) in 1984 to 186 per 10,000 (95% CI, 157-214) in 1995, during the same period in which IHD admissions in the general Ontario population decreased from a high of 101 per 10,000 in 1982 to 82 per 10,000 in 1997. Similar trends were noted for admission for MI and number of IHD events per patient during this period. The authors attributed these trends to the increasing number of IHD risk factors over time. To further investigate IHD risk factors in this vulnerable population, the Study of Health Assessment and Risk Evaluation in Aboriginal Peoples (SHARE-AP) evaluated clinical history, electrocardiography, B-mode carotid ultrasonography, and serum studies in 301 Aboriginal people from the Six Nations Reservation and 326 people of European origin from Hamilton, Toronto, and Edmonton. Compared to the Europeans, Aboriginal people had had more carotid atherosclerosis, higher rates of smoking, glucose intolerance, obesity, abdominal obesity, and inflammatory biomarkers. Aboriginal people had higher rates of unemployment and lower household income, along with higher rates of IHD risk factors within each income level. Taken together, these studies point to poverty and IHD risk factors to explain the rising rate of IHD in this population, which may contribute to the incidence of HF in this population. 

## HEART FAILURE WITH PRESERVED EJECTION FRACTION

Although most prior studies of HF have focused on those with reduced EF (i.e., systolic HF), it is now well known that HF with preserved LVEF (HFpEF) is just as common and increasing in prevalence. HFpEF is a clinical syndrome defined by signs and symptoms of HF in the absence of reduced EF [81, 82] Generally, patients with HFpEF have an EF of greater than 45-50%, depending on the study. Although several recognized specific disorders are associated with HFpEF, including restrictive cardiomyopathy, hypertrophic cardiomyopathy, infiltrative cardiomyopathy, constrictive pericarditis, and valvular heart disease, the majority of patients with HFpEF have no single distinct mechanism accounting for the syndrome. These patients typically have one or more of the following underlying processes: diastolic dysfunction due to impaired LV relaxation and/or increased LV diastolic stiffness, LV enlargement with increased intravascular volume, abnormal ventricular-arterial coupling with increased arterial and ventricular systolic stiffness, and subtle abnormalities of systolic function despite preserved EF [83-86]. 

Multivariate analysis of the Framingham Heart Study determined that compared to patients with HF and reduced EF, patients with HFpEF were older (OR 1.24 per 10 year increment, 95% CI 0.96-1.62), more likely to be female (OR 2.29, 95% CI 1.35-3.90), more likely to have atrial fibrillation at the time of HF onset (OR 4.23, 95% CI 2.38-7.52), less likely to have a left bundle branch block (OR 0.21, 95% CI 0.10-0.46), or prior MI (OR 0.32, 95% CI 0.19-0.53), and had higher systolic blood pressure (OR 1.13 for every 10 mmHg increase, 95% CI 1.04-1.22) [87]. Registry studies have revealed that HFpEF represents approximately half of acute HF presentations and has a similarly high mortality and re-hospitalization rate as in patients with HF and reduced EF [88-90]. Unlike HF with reduced EF, there are no therapies to date that have proven to improve morbidity and mortality, although there are agents under investigation [91].

Due to regional variation in risk factors for HFpEF, as well as temporal changes within regions, the prevalence of HFpEF is likely to differ between countries in North America. In the U.S. Get With the Guidelines Heart Failure program (GWTG-HF), 110,621 patients admitted from 2005-2010 were evaluated based on EF, with HFpEF having EF ≥ 50%, HF-borderline EF having 40% ≤ EF < 50%, and HF-reduced EF < 40% [92]. Overall, 36% of all patients had HFpEF, with a growing proportion of patients in this category from 2005 to 2010 from 33% to 39%, accompanied by a decrease in proportion of HF-reduced EF from 52% to 47% and stable rates of HF-borderline EF at approximately 15% (P<0.0001 for overall trend). Patients with HFpEF were older, more likely female, were more likely to have HTN, atrial fibrillation, and chronic kidney disease, had higher systolic blood pressure (SBP) and BMI, and had lower natriuretic peptides, troponin, and glomerular filtration rate than patients with HF-reduced EF. After adjustment for major covariates, patients with HFpEF were less likely to have adequate blood pressure control (OR 0.44, 95% CI 0.42-0.46), or receive HF discharge instructions (OR 0.85, 95% CI 0.80-0.89) compared to patients with HF-reduced EF. Blood pressure control at discharge was the only metric that failed to improve over between 2005 and 2010. In-hospital mortality and length of stay were similar across EF strata. However, patients with HFpEF were more likely to be discharged to a skilled nursing facility (OR 1.16, 95% CI 1.11-1.22), likely reflecting the advanced age and higher number of comorbidities in this population. This study highlighted the increasing prevalence of HFpEF in the U.S., the difficulties that persist in managing this population, especially in controlling blood pressure, and the relative frailty of this population at the time of discharge. 

Analysis of a cohort of 2,802 patients hospitalized for HF whose EF had been assessed in the Enhanced Feedback for Effective Cardiac Treatment (EFFECT) study in Ontario, Canada between 1999 and 2001 demonstrated the HFpEF (EF >50%) prevalence to be 31%, similar to that in GWTG-HF [89]. The comorbidities and presenting features of patients with HFpEF in the EFFECT study were also similar to that in the GWTG-HF study. Long-term outcomes were measured, and there was no difference in one-year mortality between patients with HFpEF and HF-reduced EF (EF < 40%, adjusted HR 1.13, 95% CI 0.94-1.36). Despite similar outcomes, assignment of a cardiologist as the primary physician and consultation with a cardiologist were lower in patients with HFpEF compared to those with HF-reduced EF (24.7% versus 33.6%, P<0.001; and 37.3% versus 43.8%, P=0.002, respectively). 

**Fig. (3):**
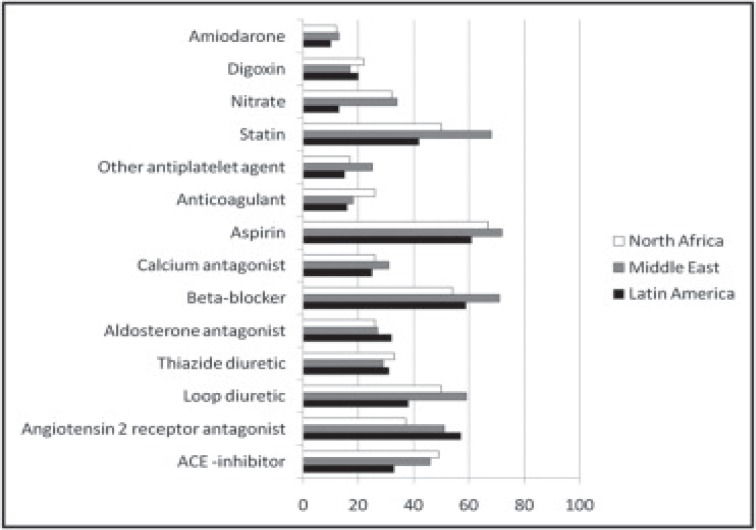
Medication use in patients with HFpEF by region. Data are percentages of the population within each region. Reprinted with permission [93].

The Identification of patients with heart failure and PREserved systolic Function: an Epidemiological Regional (I PREFER) study was a multiregional, cross-sectional, observational study across Latin America, the Middle East, and North Africa to determine the prevalence and characteristic of patients with HFpEF in these regions. [93) Unlike the GWTG-HF and EFFECT studies, the cutoff EF for HFpEF was ≥ 45% and HF-reduced EF was < 45%, and all patients had stable HF symptoms (i.e. not hospitalized for decompensated HF). Of the 868 Latin American subjects, 458 (53%) were from Mexico. Similar to other registries, patients with HFpEF were older, more likely female and obese, had higher rates of HTN and poorly-controlled BP, were more likely to have atrial fibrillation, and less likely to have prevalent CHD. Unlike other registries, the presence of valvular heart disease was measured, and there was a higher prevalence of valvular heart disease in patients with HFpEF compared to those with HF-reduced EF (32% versus 26%, P=0.005). The reported prevalence of HFpEF was higher than in other registries (65%, [95% CI 63-67%] overall; 69% [95% CI 65-72%] in Latin America), which can only be partially accounted for by a lower EF cutoff. There were important differences between regions that may explain the differences in prevalence of HFpEF. Compared to patients with HFpEF in the Middle East and North Africa, those in Latin America were older, more likely to be women, were more likely to have HTN, and valvular heart disease, and were less likely to smoke, be obese, and have DM, CHD, and atrial fibrillation. These data indicate that the predominant cause of HFpEF in Latin America may be poorly-controlled blood pressure and valvular heart disease, further supported by lower rates of loop diuretic, ACE-inhibitor, and calcium antagonist use and intermediate rates of beta-blocker use compared to the Middle East and North Africa (Fig. **3**), and higher rates of LV relative wall thickness >0.44 (66% versus 61% and 40%). It appears that HFpEF in the North America represent a group of patients with advanced age, multiple comorbidities. Uncontrolled HTN appears to be a distinguishing feature among all three countries, which remained high over time and despite measures to educate patients and control blood pressure. It will remain a challenge in all countries to understand this patient population and improve outcomes.

## ACUTE HEART FAILURE 

**Fig. (4):**
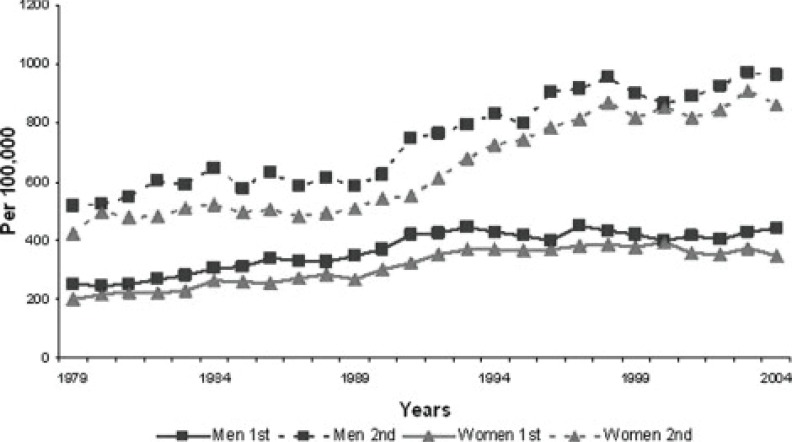
Age-adjusted hospitalization rates for acute heart failure in the United States. National Hospital Discharge Survey, 1979-2004. Reprinted with permission [5].

Acute HF (AHF), defined as a rapid onset of, or change in, signs and symptoms of HF [82]. It is often life-threatening, requiring immediate medical attention and usually leads to urgent hospital admission. AHF predominantly arises as a deterioration of patients with preexisting HF (with preserved or reduced EF) but can also be the first presentation of HF (i.e., *de novo* AHF). AHF may be precipitated by one or more clear triggers (i.e. arrhythmia, ischemia, hypertensive crisis, infection, medication non-compliance, dietary indiscretion). Presentation may vary in acuteness from days to weeks of deterioration. Hospitalization for AHF is a common and growing problem on a global scale. In the U.S., the incidence of first hospitalization for AHF is approaching 400 per 100,000 population and is approaching 1,000 per 100,000 population for the second hospitalization (Fig. **4**) [5]. In AHF, there is tremendous heterogeneity in underlying cause of HF, presence and type of comorbidities, precipitating factor for AHF, type of AHF presentation, and treatment approach. Thus AHF varies significantly across various geographic regions. This section will discuss AHF etiology, severity, management, and outcomes mostly in the U.S. and Canada. Specific data for Mexico is only available in the context of large, international studies.

### Acute Heart Failure Etiology

Etiology for AHF varies by region and follows general trends for chronic HF as discussed above. Comparison between regions was performed using the Efficacy of Vasopressin antagonism in Heart Failure: Outcome Study with Tolvaptan (EVEREST) trial, a prospective, international, randomized double-blind, placebo-controlled trial that examined the efficacy and safety of tolvaptan, a selective vasopressin-2 antagonist, in addition to optimal medical therapy in patients with reduced systolic function (EF ≤ 40%) hospitalized for worsening HF [94]. There were four distinct geographic regions in EVEREST comprising the 4,133 subjects: North America 1,251 (30.3%), South America 699 (16.9%), Western Europe 564 (13.6%), and Eastern Europe 1,619 (39.2%). Canada accounted for only 112 subjects (6.9% of North America), and Mexico was not represented. In this trial, patients in North America had the highest rates of comorbidities, including HTN, hypercholesterolemia, DM, chronic kidney disease, severe obstructive lung disease, and peripheral vascular disease, while patients in South America had the lowest rates of coronary artery disease, previous myocardial infarction, and hypercholesterolemia. These findings are consistent with known prevalence rates of comorbidities worldwide.

### Acute Heart Failure Severity

Acute HF severity can be measured using known predictors of poor outcome, such as hypotension [95], renal dysfunction, [96], hyponatremia [97], elevated biomarkers [98], respiratory distress, or concomitant comorbidities. Multivariate analysis of several variables available at the time of admission for 2,624 patients hospitalized with AHF in the EFFECT study (Ontario, Canada) was used to predict subsequent 30-day and 1-year mortality [99]. Independent predictors of 30-day and 1-year death were validated on 1,407 separate Ontario AHF patients, and included age, low SBP, elevated respiratory rate, low sodium and hemoglobin, high urea nitrogen, and presence of cerebrovascular disease, dementia, chronic obstructive pulmonary disease, hepatic cirrhosis, and cancer. In addition, a simple risk score was derived and validated using the same cohorts. 

In a separate analysis, 28,521 U.S. Medicare beneficiaries and 8,180 patients from Ontario both ≥ 65 years of age, hospitalized for AHF were compared [100]. Importantly, EF was not reported in this study, so there was a mix of patients with HFpEF, HF-borderline EF, and HF-reduced EF. Compared to U.S. patients, Canadian patients with AHF were slightly younger, more likely male, had more renal insufficiency and higher rate of prior MI but were less likely to have hypertension and diabetes. Canadian AHF patients also had lower serum sodium and hematocrit (Table **2**). When all baseline factors were considered, Canadian AHF patients had higher 30-day and 1-year mortality risk scores compared to their American counterparts (mean EFFECT risk score 93.1 versus 84.0, P<0.001 and 104.0 versus 100.8, P<0.001 respectively), indicating a higher severity of illness. The authors attributed increased HF severity on presentation to the relatively lower number of hospital and intensive care unit beds available in Canada relative to the U.S.. 

In the EVEREST trial, compared to other world regions (including Western Europe, Eastern Europe, and South America), North American patients were older (second only to Western Europe), had the lowest systolic blood pressure, highest blood urea nitrogen, highest BNP, and highest rates of comorbidities. These data suggest that North American AHF patients had the most severe AHF at the time of presentation [94]. 

### Acute Heart Failure Management

In the aforementioned study comparing U.S. Medicare patients with similar-aged Canadian patients from Ontario, Canada, length of stay was longer for AHF patients in Canada than in the U.S. (8.5 versus 6.1 days, P<0.001), and Canadians underwent fewer cardiovascular procedures during AHF hospitalization, including EF assessment, cardiac catheterization, and revascularization compared to U.S. patients [100]. Use of aspirin at discharge was similar (40.0% versus 39.7%, P=0.70); however, beta-blocker and lipid-lowering agent use was slightly lower (25.4% versus 28.7% [P<0.001] and 15.0% versus 16.7% [P<0.001] respectively) in Canadian versus U.S. AHF patients, whereas ACE-inhibitor/ARB use was higher among Canadian patients (68.9% versus 62.2%, P<0.001) (Table **2**). In the EVEREST study, beta-blocker use at discharge was much higher across all continents studied (ranging from 63% to 82%), and highest in North America, as were lipid-lowering agents. However, ACE-inhibitor/ARB use at discharge was lowest in North America compared to other regions.. The use of percutaneous and surgical revascularization prior to hospitalization was highest in North America, up to 3-4 times higher than in Eastern Europe and South America. 

The comparison of discharge medication use between the registry data and the EVEREST data highlights the prescription of beta-blockers for systolic HF compared to HFpEF. Analysis of 11,854 patients ≥ 65 years of age in Alberta, Canada admitted for *de novo* AHF irrespective of EF, demonstrated a gradual increase in the use of beta-blockers from 1994 to 2000, and the combination of both beta-blockers and ACE-inhibitors and ARBs was associated with substantial improvements in one-year mortality compared to patients on neither agent (16.3%, [95% CI 12.3-20.3%] versus 29.9%, [95% CI 28.8-31.0%]) [101]. 

### Acute Heart Failure Outcomes

In the comparison of U.S. Medicare patients with similar-aged Ontario patients with AHF, unadjusted mortality rates were lower in the U.S. at 30 days (8.9% versus 12.2%, P<0.001) and at 1 year (32.2% versus 35.7%, P<0.001) compared to Canada [100]. Thirty-day mortality rates were lower for U.S. patients compared with Canadian patients (8.9% [95% CI 8.6-9.3%] versus 10.7% [95% CI 10.1-11.3%]); however, 1-year mortality was similar (32.2% 95% CI 31.7-32.7% versus 32.3% 95% CI 31.4-33.2%), after adjusting for baseline risk score. The authors suggest that the shorter hospital stay and greater use of inpatient diagnostic and therapeutic procedures may translate to better short-term mortality in the U.S., [102] while better outpatient follow-up and access to medication may explain the “catch up” in long-term mortality in Canada [103]. 

**Fig. (5):**
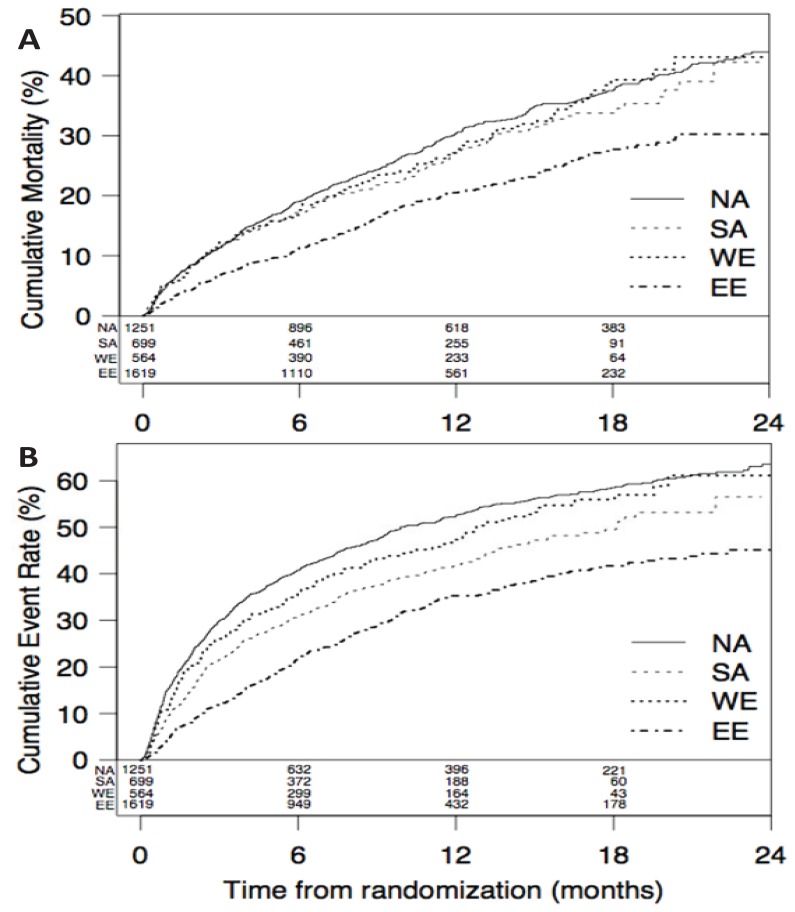
Kaplan-Meier estimates of mortality (Panel A) and combined cardiovascular death and heart failure hospitalization (Panel B) in subjects hospitalized for worsening heart failure and depressed left ventricular ejection fraction, across regions in Efficacy of Vasopressin Antagonism in Heart Failure: Outcome Study with Tolvaptan (EVEREST). Abbreviations: NA, North America; SA, South America; WE, Western Europe; EE, Eastern Europe. Reprinted with Permission [94].

In the EVEREST study, after a median follow-up of 9.9 months post-discharge, unadjusted 1-year Kaplan-Meier estimates of mortality were highest in North America (30.4%) compared to other world regions (20.5-27.2%, Fig. **5**) [94]. A similar trend was found for 1-year combined CV death/HF hospitalization rates: highest in North America (52.5%) compared to other world regions (35.3-47.3%). However, after adjusting for baseline variables (as an indicator of baseline disease severity), overall mortality and morbidity were similar in North America and Western Europe, while outcomes were worse in South America and better in Eastern Europe (HR 1.42, [95% CI 1.15-1.76] and HR 0.84, [95% CI 0.73-0.97], respectively, compared to North America). Data from the EVEREST trial highlights the impact of comorbidities on outcomes in AHF—while patients in North America have more comorbidities and more severe HF presentation than other regions, overall outcomes for these patients are similar to Western Europe when controlling for baseline risk factors. Outcomes in South America were worse than North America despite intermediate risk profile, highlighting that either HF care or unmeasured variables account for these outcomes.

## THE ECONOMIC BURDEN OF HEART FAILURE

HF is among the most costly chronic illnesses in developed countries. Comparison of costs and healthcare expenditures across countries revealed that in 2000, the United States spent approximately $23 billion, or 1.5% of total health care expenditures, on HF, with the majority of the cost attributable to hospitalization, with a similar percentage expenditure in France, the United Kingdom, New Zealand, and Sweden [104]. Detailed analysis of the British National Health System revealed that healthcare expenditure was primarily from inpatient care (69%), followed by drug treatment (18%) and outpatient visits and referrals (13%), but these estimates did not include secondary admissions and long-term nursing home care [105]. The burden of HF admissions falls heavily on patients ≥ 65 years of age [5]. With the projected number of Americans aged >65 years from 34.7 million in 2000 to 78.9 million in 2050 [106], resulting in a projected increase the incidence of HF hospitalizations by 1-1.5 million cases [107], and an exponential growth of healthcare costs for HF. In addition, temporal trends in hospital re-admission and proliferation of diagnostic testing in North America may further increase the financial burden of HF. This section will discuss the costs of the many aspects of HF care in the U.S. and Canada. There are no such data available for Mexico.

### Inpatient Care

Inpatient care is responsible for the vast majority of HF expenses. Among U.S. Medicare beneficiaries discharged from a hospital between 2003-2004, HF was the most common discharge diagnosis for patients re-hospitalized within 30 days [108]. After one admission for HF, elderly Americans have a 23% rate of re-hospitalization for HF, and 49% rate of rehospitalization for any reason within six months [109]. Similar data for Canadian HF discharges of all ages reported a nearly 50% HF readmission rate at one year [110]. 

One method to reduce cost of hospital stay may be by shortening the length of stay. Analysis of 6.96 million U.S. Medicare hospitalizations for HF revealed a decrease in the median from 8.81 days to 6.33 days, in-hospital and 30-day mortality decreased from 8.5% and 12.8% to 4.3% and 10.7%, respectively from 1993 to 2006 [111]. During this same time period, 30-day readmission rates increased from 17.2% to 20.1%, and discharge to skilled nursing facilities (SNFs) increased from 13.5% to 19.9%. From this analysis, it is not clear that from a patient or cost of care perspective if inpatient HF care in 2006 was markedly better than in 1993 in the U.S. as hospital length of stay and short-term mortality decreased but morbidity, mortality, and cost shifted to outside the hospital. In Canada, length of stay for HF is higher than that in the U.S., [100] and longer length of stay has been validated as a multivariate predictor of poor outcome in Canadian AHF populations [112, 113]. Further investigation on whether longer hospital stays with a focus on improved pharmacotherapy and addressing of comorbidities will translate into better outcomes and cost savings. 

### End-of-Life Care

Much of the inpatient healthcare expenditures occur at the end of life. In a retrospective cohort study analyzing resource use in the last 180 days of life, of the 229,543 U.S. Medicare beneficiaries with HF who died between 2000-2007, approximately 80% were hospitalized in the last 6 months of life [114]. During this time the mean number of days in the intensive care unit rose from 3.5 to 4.6 (P<0.001), use of hospice increased from 19.0% to 38.1% (P<0.001), but the mean length of stay of the final hospitalization remained approximately 20.7 days between the years 2000 and 2007. Despite the increased use of hospice, rates of other services such as physician visits did not change, while rates of echocardiography, durable medical equipment, home health, and skilled nursing facilities increased, and unadjusted costs increased from $28,766 to $36,216, indicating that the cost-saving potential for hospice has yet to be realized. During this period, overall rate of HF hospitalization decreased from 16.3% to 14.8% (P<0.001). A similar analysis of 33,144 residents of Alberta, Canada ≥ 65 years of age with HF who died between the years 2000 and 2006, was performed [115]. During this period, the proportion of patients hospitalized during the last 6 months of life decreased from 84.0% to 76.2% (P<0.001). The mean number of inpatient days stayed the same at 34-35 (P=0.90), although the mean number of days in the intensive care unit decreased from 2.3 to 1.9 (P<0.001). The percentage dying in the hospital decreased from 60.4% to 54.0% (P<0.001), despite the limited availability of hospice services in Canada. The cost to the Canadian system increased from $25,069 to $27,983 in Canadian dollars, which remained significant after multivariate adjustment. Based on these two analyses, end-of-life care is both costly and challenging in the different healthcare systems. 

### Long-Term Care Facilities

Nursing homes, skilled nursing facilities (SNFs), extended care facilities, and custodial care facilities are all types of long-term facilities that can be utilized among patients discharged for HF. Long-term care is not traditionally accounted for in HF health care cost analyses, however they make up a large part of the overall cost for patients hospitalized for HF. According to analysis of the U.S. National Discharge Survey data, the proportion of patients discharged to long-term facilities has increased for patients hospitalized for HF as a primary or secondary diagnosis from 6.8% and 8.9% in 1980-1984 to 13.4% and 21.6% in 2000-2004 [5]. A cross-sectional analysis of eight long-term facilities and 1,223 residents in Canada revealed a prevalence of HF of 20% [116].

The U.S. GWTG-HF data was linked with the Centers for Medicare and Medicaid Services claims data to determine the characteristics and outcomes of patients hospitalized for HF who were subsequently discharged to a SNF [117]. Of the 15,459 patients studied, 3,727 (24.1%) were discharged to a SNF. After multivariate analysis, patients discharged to SNFs were older, more likely female, had more medical comorbidities including stroke and depression, were less likely to have MI, revascularization or valvular heart disease, had higher EFs, higher urea nitrogen, and were less likely to have an implantable cardioverter-defibrillator (ICD), and a longer length of stay. Post-discharge mortality and rehospitalization rates were higher for patients discharged to SNFs compared to patients discharged elsewhere (1-year mortality 53.5% versus 29.1%, respectively; P<0.0001, 1-year rehospitalization 76.1% versus 72.2%, respectively; P<0.0001) associations that remained significant after adjustment for major covariates. 

In this particularly high-risk population, it is likely that patients discharged to SNFs have less optimal care than other outpatients. An analysis of 1,223 Canadian SNF residents with HF demonstrated that of the 55% of patients who were receiving ACE inhibitors, only 45% received guideline-based doses, while only 25% were receiving beta-blockers [116]. Improving mortality rates for SNF residents may lie in initiation of evidence-based therapies at the time of discharge and adequate follow-up for titration of these medications and/or evaluation for ICD placement. Conversely, there may be a subset of HF discharges intended for SNF at particularly high risk for death and/or rehospitalization. Use of prognostic scores such as the EFFECT and LACE (Length of stay, Acuity of admission, Charlston comorbidity index score, Emergency department use) risk scores developed in Canada have promise identifying high-risk patients who may benefit from adequate counseling on the expectations of survival and rehospitalization, deliberation of alternatives to SNF including hospice, and formal consideration of overall goals of care and code status [112, 113, 118]. It will remain a challenge to ensure that these high-risk patients receive adequate ongoing care for HF in order to prevent rehospitalization and death.

### Outpatient Care:

Total outpatient costs include outpatient visits, emergency department visits, medications, outpatient procedures, and diagnostic testing and make up 20-30% total costs of HF care [105]. A substantial number of patients with HF-reduced EF are not treated with ACE-inhibitors and beta-blockers, or are not receiving optimal doses [119]. Analysis of the OPTIMIZE-HF study demonstrated that 61.3% of patients hospitalized for HF had one or more identifiable precipitating factors, several of which (uncontrolled hypertension [10.7%], nonadherence to medications [8.9%], and nonadherence to diet [5.2%]) may have been prevented in the outpatient setting [11]. Analysis of GWTG-HF data demonstrated that the 10.3% of patients nonadherent with either medication or diet had a lower in-hospital mortality and length of stay despite a higher risk profile, indicating that these patients may be easier to stabilize and may be easier to prevent from being hospitalized [120]. In order to reduce costs and improve morbidity and mortality, visits should focus on evidence-based therapies for HF, as well as strategies optimization of comorbidities and potential triggers for admission.

HF disease management (HFDM) programs have emerged as a method to reduce rehospitalization and improve quality and cost-effectiveness for selected HF patients by optimizing treatment of HF comorbidities and precipitants, and through patient education on adherence to evidence-based medications and fluid/sodium restriction. A meta-analysis of 11 randomized clinical trials involving 2,067 patients with HF demonstrated that hospitalizations (RR 0.87, [95% CI 0.79-0.96]) but not all-cause mortality (RR 0.94, [95% CI 0.75-1.19]) were reduced by the programs, which seemed to be driven by the effect of specialized follow-up by a multidisciplinary team (RR 0.77, [95% CI 0.68-0.86]) versus trials with telephone contact or improved coordination with primary care services (RR 1.15, [95% CI 0.96-1.37]) [121]. HFDM programs appear to be even more effective when used in conjunction with comprehensive post-discharge planning after admission for HF. Another meta-analysis evaluated 18 randomized controlled trials including patients ≥ 55 years of age testing interventions intended to modify hospital discharge for HF and provide post-discharge support [122]. Patients in the comprehensive discharge/HFDM group had lower rehospitalization rates (RR 0.75, [95% CI 0.64-0.88]), a trend towards lower all-cause mortality rates (RR 0.87, [95% CI 0.73-1.03]), significant improvement in quality of life scores, and a trend towards cost savings (-$359, [95% CI $-763 to $45]).

Despite several clinical trials demonstrating improved clinical and financial outcomes, there have been examples where healthcare organizations in the U.S. have successfully initiated HFDM programs but withdrew them over time due to the existing reimbursement structure [123]. A financial model using another meta-analysis was developed to compute the expected costs before and after implementation of a HFDM program stratified by three provider types (physicians, hospitals, and health systems) and costs incurred from a payer perspective [124]. This analysis showed that the implementation of HFDM results in a net loss to all provider types, with the highest impact on hospitals. Although there are significant savings for the payer perspective, there is not enough incentive to start and/or maintain such programs in the current reimbursement system in the U.S., outside of healthcare management organizations and academic centers. 

The universal health care system in Canada provides unique opportunity for implementation of HFDM programs. A comparative analysis of claims from all elderly individuals in the three largest Canadian provinces using data from provincial ministries of health, and a 1% random sample of U.S. elderly Medicare beneficiaries not enrolled in health maintenance organizations from the U.S. Health Care Financing Administration in 1992 demonstrated that Canadian elderly receive 44% more evaluation and management services but 25% fewer procedures than their U.S. counterparts [103]. The lower price for physician services in Canada and relative scarcity of diagnostic testing make Canada an ideal country for widespread use of HFDM programs. There is evidence of robust networks of HFDM programs in Canada, as observed by the growth of HF clinic groups between 1998 and 2002 in Nova Scotia [125]. Restructuring of the incentive system in the U.S. through programs such the Center for Medicare and Medicaid Service’s Readmissions Reduction Program [126] may make HFDM programs more attractive for hospital systems, allowing better utilization of these programs, similar to the way Canada’s single-payer structure does.

### Medications

Review of cost-effectiveness is reviewed elsewhere; however, several trials have established that ACE-inhibitors, beta-blockers, digoxin, and spironolactone are cost-saving medications, mostly through their reduction in hospitalization [127]. It appears that the use of ACE-inhibitors and beta-blockers is widespread in patients hospitalized for HF and increases at the time of discharge. Analysis of patients with HF-reduced EF (<40%) in the GWTG-HF program reported an impressive rate of 65.3% ACE-inhibitor or ARB use at admission and 92.9% at discharge and a rate of 72.6% beta-blocker use at admission and 94.6% at discharge in patients eligible for medical therapy without contraindication [128]. Current data are not available for Canada, although comparison of elderly patients hospitalized for HF demonstrated lower use of ACE-inhibitors and beta-blockers in both Canada and the U.S., presumably due to inclusion of patients with HFpEF in the analysis and lack of efficacy of these medications in such patients (Table **2**) [100].

Although the prescription of evidence-based medications in patients hospitalized for HF and reduced EF is excellent in the U.S. and likely Canada, patient adherence to these medications differs between countries. Analysis of prescription-filling patterns in 54,153 U.S. Medicare beneficiaries with at least one hospitalization for HF (with no EF-based exclusion criteria) between 1995 and 2003 revealed that only 49%, 29%, and 5% filled prescriptions for ACE-inhibitors/ARBs, beta-blockers, and spironolactone, respectively within 90 days of HF hospitalization, and optimal adherence (≥ 80% adherence) to these medication was <55% [129]. There were only modest increases in adherence over time for beta-blockers and spironolactone, and no significant change over time for ACE-inhibitors/ARB. There is evidence that expanded medication coverage may improve this situation. A separate study analyzed pharmacy claims for 6,950 patients with HF ≥ 65 years of age enrolled in the a large health insurer in Pennsylvania two years before and after implementation of Medicare Part D (2003-2007) [130]. Prescription fill patterns among patients who moved from limited or no drug coverage to Part D with those who had employer-sponsored coverage throughout the study revealed that those patients who switched from no coverage to Part D were more likely to fill prescriptions for ACE-inhibitors/ARBs plus beta-blocker (adjusted OR 1.73, 95% CI 1.42-2.10) and more likely to adhere to their regimen (adjusted OR 2.95, 95% CI 1.85-4.69) compared to those with employer-sponsored coverage. 

Canadian trends in medication adherence after HF hospitalization appear better than those in the U.S. Review of administrative medical databases in Saskatchewan, Canada demonstrated that of the 8,805 patients discharged from the hospital with a primary diagnosis of HF who survived at least one year after discharge between 1994 and 2003, 5% filled a prescription for a beta-blocker within 6 months of discharge in 1994/1995, which increased to 32% in 2002/2003 [131]. Mean 1-year percentage of patients exhibiting optimal adherence rate improved for both beta-blocker and ACE-inhibitor/ARBs from 71% and 80% in 1994/1995 to 83% and 88% in 2002/2003, respectively. Since prescription writing and dispensation policies remained unchanged during the study period in Saskatchewan, the authors hypothesized that improved management of HF patients over time drove adherence. It remains to be seen whether expanded medication coverage will result in widespread improvement in medication adherence in the U.S. and whether these changes will result in improved outcomes for patients with HF in both the U.S. and Canada on a population level.

### Procedures

The utilization and proliferation of cardiac procedures such as echocardiography, stress testing, cardiac catheterization, and percutaneous coronary intervention (PCI) is on the rise in both the U.S. and Canada, although absolute rates of utilization are lower in Canada. Cross-sectional population-based studies in the U.S. and Canada between 1992 and 2001 demonstrated year-over-year increases in cardiac testing that outstripped the rate of MI in both countries [132, 133]. These studies highlight the proliferation of cardiac technology in both countries. As echocardiography and coronary testing are common tests in HF populations, treatment for HF undoubtedly has contributed to the use of these procedures. Both nations face significant challenges in containing costs while providing quality care in HF patients who are being considered for cardiac procedures.

## UNANSWERED QUESTIONS AND FUTURE CHALLENGES

HF, a complex clinical syndrome which represents the culmination of a variety of cardiovascular disease processes, remains a global public health problem. HF is particularly common and costly in North America, and the prevalence of HF is projected to grow as the population ages, risk factors such as diabetes and obesity continue to rise, and as survival from cardiovascular conditions such as MI increases. There are several questions that remain unanswered and require further investigation. First, there is a lack of data on HF in Mexico and how it compares to the U.S. and Canada. Understanding this relationship may lead to better HF prevention and care in Mexico and for Mexican patients in the U.S. and Canada. Second, since HF is a heterogeneous condition whose etiology, severity, and management varies across regions, it remains to be determined whether the results of a particular clinical trial for new HF therapies applies across all countries. Third, it remains to be determined whether nation-wide public health initiatives to control risk factors for HF, such as the Canadian Hypertension Education Program, salt reduction, and metrics to improve MI care, translate into improved HF incidence and outcomes. Fourth, throughout the world and in North America, HFpEF continues to be a major problem because of heterogeneity of the HFpEF syndrome, lack of effective therapies, and its rising prevalence in the population. Thus, future research should focus on this difficult patient population. Fifth, AHF represents a major clinical challenge, both because of the absence of effective evidence-based therapies and the alarmingly high readmission rates after hospitalization for HF. Canada has taken initiatives to develop extensive disease management programs in an effort to reduce AHF hospitalizations, but such strategies have yet to be proven financially viable in the U.S. Finally, the cost of HF management has escalated rapidly for treatment AHF inpatients in North America at a time when cardiac diagnostic testing use is also increasing; thus, development of novel payment/incentive structures are necessary to help expand cost-saving measures to reduce overall costs of HF management without sacrificing quality. Many challenges lie ahead in the global mananagement of the HF epidemic. Progress has been made in understanding the epidemic in North America that has the potentential to yield improvements in HF prevention, management, and outcomes. 

## Figures and Tables

**Table 1.** Population attributable risk of heart failure due to risk factors in the United States in 5,545 men and 8,098 women in the National Health and Nutrition Examination Survey (NHANES)-I Epidemiologic Follow-up Study[9]

Risk Factor	Adjusted Relative Risk (RR, 95% Confidence Interval)	P-value, RR	Population Attributable Risk, %
Coronary Heart Disease	8.11 (6.95-9.46)	<0.001	61.6
Cigarette Smoking	1.59 (1.39-1.83)	<0.001	17.1
Hypertension	1.40 (1.24-1.59)	<0.001	10.1
Low Physical Activity	1.23 (1.09-1.38)	<0.001	9.2
Male Gender	1.24 (1.10-1.39)	<0.001	8.9
Less than High School Education	1.22 (1.04-1.42)	0.01	8.9
Overweight	1.30 (1.12-1.52)	0.001	8.0
Diabetes	1.85 (1.51-2.28)	<0.001	3.1
Valvular Heart Disease	1.46 (1.17-1.82)	0.001	2.2

**Table 2.** Baseline characteristics of 28,521 American and 8,180 Canadian patients ≥ 65 years of age with acute heart failure. Ab-breviations: SD, standard deviation; LVEF, left ventricular ejection fraction; ACE, angiotensin converting enzyme; ARB, angiotensin receptor blocker. Adapted from [100]

Characteristic	United States	Canada	P-value
**Demographic**			
Age, mean (SD), y	80.1 (7.7)	79.7 (7.5)	<0.001
Female, %	57.3	45.2	<0.001
**Physical and laboratory findings**			
Systolic blood pressure, mean (SD), mmHg	149.2 (30.9)	148.6 (33.2)	0.11
Urea nitrogen, mean (SD), mg/dL	27.5 (16.9)	29.8 (18.8)	<0.001
Renal insufficiency (creatinine >2.5 mg/dL or urea nitrogen >40 mg/dL), %	15.8	19.2	<0.001
Serum sodium, mean (SD), mol/L	138.6 (5.0)	138.3 (4.9)	<0.001
Hematocrit, mean (SD), %	37.7 (6.0)	36.9 (6.1)	0.02
**Medical history, %**			
Hypertensin	62.3	48.3	<0.001
Diabetes	34.1	32.0	<0.001
Previous myocardial infarction	25.6	36.0	<0.001
Cerebrovascular disease	17.4	17.6	0.74
Dementia	9.9	9.2	0.06
**Mortality risk score, mean**			
30-day	84.0	93.1	<0.001
1-year	100.9	104.0	<0.001
**In-hospital care and procedures**			
Length of stay, mean (SD), d	6.1 (4.4)	8.5 (12.3)	<0.001
Cardiologist as attending physician, %	18.8	19.4	0.22
LVEF assessment, %	61.2	41.7	<0.001
Cardiac catheterization, %	5.6	0.59	<0.001
Percutaneous coronary intervention, %	0.57	0.05	<0.001
Coronary artery bypass grafting, %	0.41	0.04	<0.001
**Discharge medications, %**			
Aspirin	39.7	40.0	0.70
Beta-blockers	28.7	25.4	<0.001
ACE inhibitors or ARBs	62.2	68.9	<0.001
Lipid-lowering medications	16.7	15.0	<0.001
